# Horizontal cortical connections shape intrinsic traveling waves into feature-selective motifs that regulate perceptual sensitivity

**DOI:** 10.21203/rs.3.rs-3830199/v1

**Published:** 2024-01-09

**Authors:** Zachary W. Davis, Alexandria Busch, Christopher Stewerd, Lyle Muller, John Reynolds

**Affiliations:** 1 The Salk Institute for Biological Studies, La Jolla, CA, USA. 92037; 2 Department of Ophthalmology and Visual Science, University of Utah, SLC, UT, USA 84112; 3 Department of Applied Mathematics, Western University, London, ON, Canada. N6A 3K7; 4 Brain and Mind Institute, Western University, London, ON, Canada. N6A 3K7

## Abstract

Intrinsic, ongoing fluctuations of cortical activity form traveling waves that modulate the gain of sensory-evoked responses and perceptual sensitivity. Several lines of evidence suggest that intrinsic traveling waves (iTWs) may arise, in part, from the coordination of synaptic activity through the recurrent horizontal connectivity within cortical areas, which include long range patchy connections that link neurons with shared feature preferences. In a spiking network model with anatomical topology that incorporates feature-selective patchy connections, which we call the Balanced Patchy Network (BPN), we observe repeated iTWs, which we refer to as *motifs*. In the model, motifs stem from fluctuations in the excitability of like-tuned neurons that result from shifts in E/I balance as action potentials traverse these patchy connections. To test if feature-selective motifs occur *in vivo*, we examined data previously recorded using multielectrode arrays in Area MT of marmosets trained to perform a threshold visual detection task. Using a newly developed method for comparing the similarity of wave patterns we found that some iTWs can be grouped into motifs. As predicted by the BPN, many of these motifs are feature selective, exhibiting direction-selective modulations in ongoing spiking activity. Further, motifs modulate the gain of the response evoked by a target and perceptual sensitivity to the target if the target matches the preference of the motif. These results provide evidence that iTWs are shaped by the patterns of horizontal fiber projections in the cortex and that patchy connections enable iTWs to regulate neural and perceptual sensitivity in a feature selective manner.

## Introduction

Even in the absence of external input, cortical neurons are spontaneously active^1–3^. This spiking activity emerges from the highly recurrent and interconnected anatomical organization of cortical networks^2–6^. The majority of these connections are local to a cortical area with neurons receiving roughly 80% of their synaptic inputs through horizontal fibers^7–9^. Correlated synaptic activity gives rise to large fluctuations in network state that can be measured in the local field potential (LFP)^10–13^. Across multiple cortical areas, LFPs have been shown to exhibit diverse spatiotemporal patterns including traveling waves^14^. Traveling waves of neural activity have been observed in the occipital^15–23^, parietal^24–27^, temporal^28,29^, and frontal lobes^24,30^, as well as the insula^31^ and hippocampus^31,32^. They have been linked to fluctuations in cortical excitability that impact sensory processing and perception^18^, motor reaction time^26,33^, memory function^30^, and reward anticipation^24^. While traveling waves can be generated from external stimuli or motor behaviors including saccades^17,21,23,34^, they also occur spontaneously. We refer to these spontaneous waves as *intrinsic traveling waves* (iTWs)^18^.

Given the ubiquity of iTWs across cortical systems and the diversity of behavioral contexts in which they appear to play a role, it is of interest to understand the neural mechanisms that give rise to them and mediate their impact on cortical computation. We have proposed a theoretical model in which the spiking activity of neurons naturally takes the form of iTWs^35^. The model shows that iTWs occur spontaneously in the spiking activity of sufficiently large scale networks of neurons (100,000’s of neurons interconnected by 100,000,000s of synapses) with distance-dependent connectivity, axon conduction velocities matching those of unmyelinated horizontal fibers^36,37^, and synaptic conductances consistent with those measured *in vivo*^38*–*40^. The iTWs that arise spontaneously in the model mirror statistical properties of iTWs we observe *in vivo*, including their speed of propagation, wavelength distribution, and frequency of occurrence^18,35^. In addition to accounting for how iTWs may form, the model also explains how iTWs may mediate observed changes in the gain of stimulus evoked responses. In the model, fluctuating synaptic inputs drive dynamic shifts in E/I balance that travel as waves through topographic anatomical connectivity, as measured through a model of the LFP^13,41,42^. When a relatively depolarized wave state aligns with the onset of a sensory input, they yield stronger evoked responses. Finally, the model explains observed changes in perceptual sensitivity that accompany iTWs, with iTW-dependent elevations in the gain of the stimulus-evoked response driving increases in perceptual sensitivity.

However, while the model is able to account for many aspects of cortical iTWs with only local Gaussian connectivity, it does not incorporate a prominent anatomical feature of long-range horizontal fibers: patchy feature-selective connectivity^43^. In multiple species and cortical areas including V1^44–46^, V2^47–49^, MT^47,50^, IT^51,52^, PFC^53^ and motor cortex^54^, unmyelinated horizontal fibers are anisotropic, bypassing intervening tissue to connect patches of neurons that share feature preferences in common. The presence of these projections might offer an additional test of the proposition that iTWs, as measured in the LFP, reflect underlying spatiotemporal patterns of spiking activity as they traverse the horizontal fiber network. In particular, we reasoned that action potentials traversing these feature-selective horizontal fiber connections might momentarily shift the E/I balance among neurons that share feature preferences in common, leading to feature-selective regulation of spiking activity and perceptual sensitivity.

To formalize this intuition, we added long-range feature-selective projections to the model to generate patchy connectivity as observed *in vivo*. We call this model the Balanced Patchy Network (BPN) as it is an anatomically organized extension of standard balanced random networks^55–57^. Using newly developed tools to compare iTWs, we find distinct wave patterns that tend to repeat in the ongoing activity of the BPN. We refer to these repeating wave patterns as *motifs.* Motifs have previously been observed in cortical activity, often using widefield optical imaging^58–62^ and are thought to reflect common activation patterns due to the organization of anatomical connectivity^58^. However, their function remains unclear.

In the BPN, we find that wave motifs are associated with feature-specific modulations in spiking activity across the network. We tested if feature-selective motifs can be observed in the brain by examining iTWs that we had previously collected using Utah multielectrode arrays placed in marmoset Area MT while the monkeys performed a threshold visual detection task^18^. We find, consistent with model predictions, that some iTWs can be classified into recurring motifs that, according to the model, drive modulations in undriven spiking activity among like-tuned neurons. We then asked whether these feature-selective motifs impact the monkey’s performance on the detection task. We find that when an iTW that is a member of a motif occurs at the time of the target-evoked response, this modulates the gain of evoked responses and the likelihood of the marmoset detecting the target, provided the target’s features match the preference of the motif. These findings provide evidence that iTWs are indeed shaped by the anatomical patterns of connectivity of the cortex and introduce a novel computational role for the long-range patchy connections that are found in the neocortex: namely that they drive feature-selective changes in spiking activity and perceptual sensitivity.

## Results

### The Balanced Patchy Network

**Figure 1.**
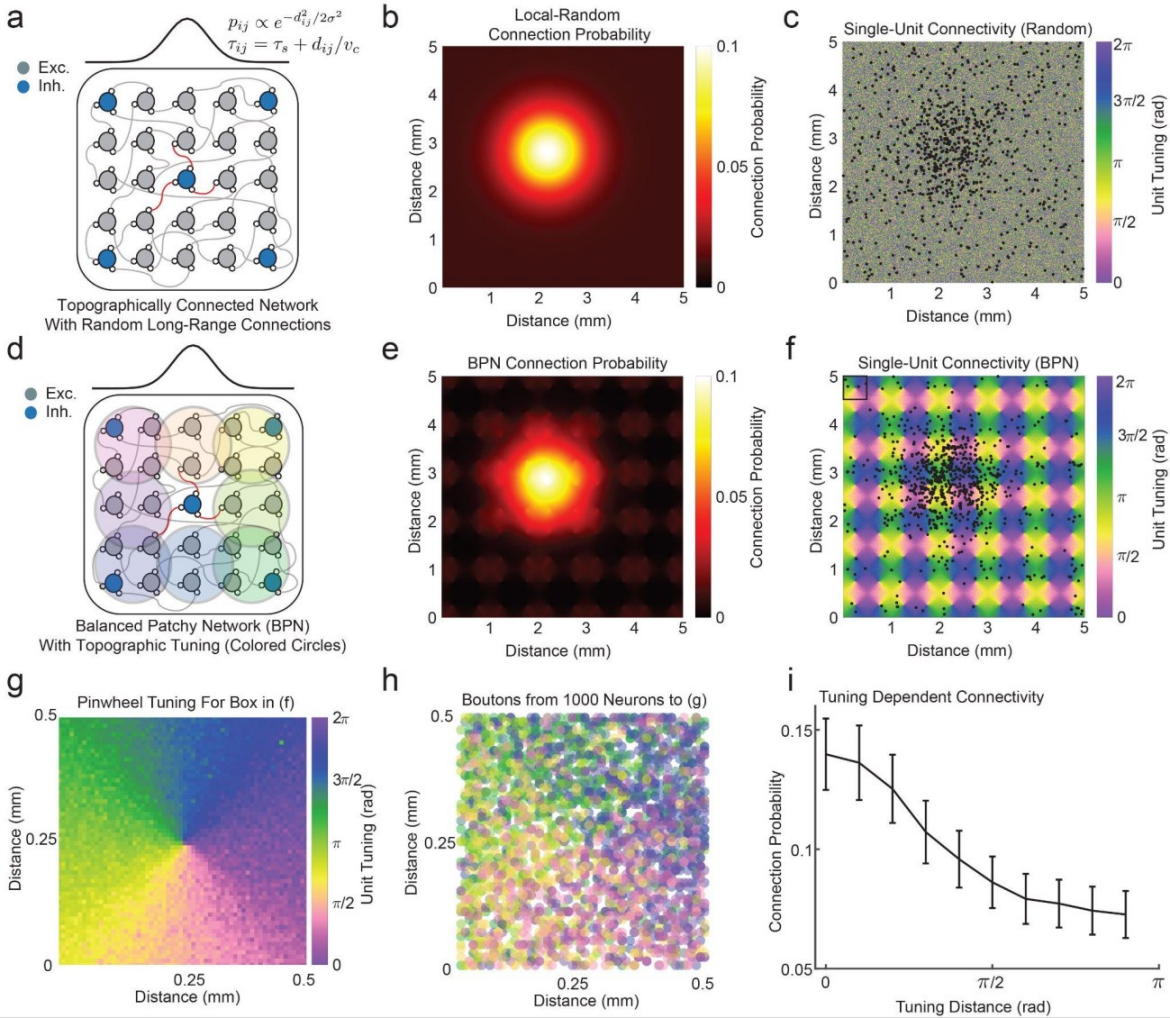
Tuning-dependent long range connectivity in the Balanced Patchy Network. (a). Schematic of previously published spiking network model with 90 percent local and 10 percent random long-range connectivity with distance-dependent time delays. (b). Network connection probability for an example unit in the random long-range model. (c). Black dots show the actual synaptic boutons for the example unit in b with random tuning preferences across the network. (d) Schematic of BPN with tuning-dependent patchy long range connectivity. Colored circles indicated topographic regions with similar tuning preference. (e) Connection probability for the BPN with tuning-dependent long-range probabilities. (f) Synaptic boutons from the single unit in (e) form patchy connections with like-tuned neurons in the BPN. (g) Tuning topology of single pinwheel (box in f). Each pixel is a neuron color coded by its assigned tuning preference. (h) Boutons formed by long-range projections (> 2 mm) from 1000 neurons to the neurons in (g), color coded by the projecting neuron’s tuning preference. Connections preferentially tile according to the pinwheel topology in g. (i). Total connection probability from both local and long-range connectivity rules is strongly dependent on tuning distance between pairs of neurons (error bars are the standard deviation across N = 10000 random excitatory neurons).

To test the hypothesis that long-range patchy connections give rise to feature-selective iTWs, we modified a previously published spiking network model that recapitulates properties of iTWs observed Area MT of the common marmoset (Figure 1a). This model consists of 450,000 leaky integrate-and-fire neurons (80 percent excitatory, 20 percent inhibitory) arranged as a 2 dimensional network 5 × 5 mm in area. Each neuron makes 1,000 conductance-based synaptic connections and the locations of these connections were determined probabilistically with 90 percent drawn from a Gaussian distribution (600 μm sigma) and the remaining 10 percent of synapses distributed at random across all neurons. (Figure 1b, c). Synaptic activations occur after a delay proportional to the distance between each pair of neurons to reflect the conduction delays of action potentials propagating along unmyelinated horizontal cortical fibers (velocity = 0.2 m/sec). When excitatory and inhibitory synaptic conductances are appropriately balanced, this network generates locally asynchronous-irregular spiking dynamics that give rise to iTWs without the need for any external driving input^56,57,63,64^. The statistical properties of the model (spike rate distribution, coefficient of variation of spiking activity, iTW wavelength and iTW propagation speed) are similar to spiking and LFP data recording *in vivo*^35^. The model also recapitulates the experimentally observed phase dependence of undriven spiking activity and stimulus-evoked response gain^35^.

This previously published model did not include a prominent anatomical feature that has been observed in multiple neocortical areas: that unmyelinated horizontal fibers are anisotropic, bypassing intervening tissue to connect patches of neurons that share feature preferences in common. To achieve this connectivity pattern in the BPN, we assigned tuning preferences to the neurons in the network with a topology approximating the motion direction tuning pinwheels seen in primate Area MT^65–67^. The 10% long-range connections were preferentially formed among neurons with similar tuning for motion direction, with the connection probability between neurons falling off with the circular distance between the neurons’ tuning preference (modeled as a von Mises distribution) independent of spatial distance (Figure 1d, e). This resulted in a connectivity map where most of the connections were local, as in the original network model, but the long-range connections formed patches among neurons assigned similar tuning preference (Figure 1f). The consequence of this tuning rule is that, within an individual pinwheel (Figure 1g), synaptic contacts from distant sources are clustered according to the shared tuning preference of the synaptic partners (Figure 1h). The total probability of connectivity from both the local and long-range connections combined are thus dependent on the difference in tuning preference between neurons (Figure 1i).

### Balanced Patchy Network Predictions

We hypothesized that the incorporation of patchy, feature-selective connections would, through the delays from spikes traversing the long-range projections, give rise to iTW patterns reflecting the shared synaptic activity among the neurons within a feature-selective network. We predict that when a particular subnetwork is more active, this will produce a particular iTW pattern and similar patterns will occur if similar subnetworks are active. We refer to the patterns common to groups of similar iTWs as *motifs*. Different motifs will then correspond to different subnetworks being active during the iTWs, and because the neurons that are linked through long-range patchy connections share feature preferences with one another, we predict that iTWs within a motif will coincide with feature-selective regulation in spiking activity.

**Figure 2.**
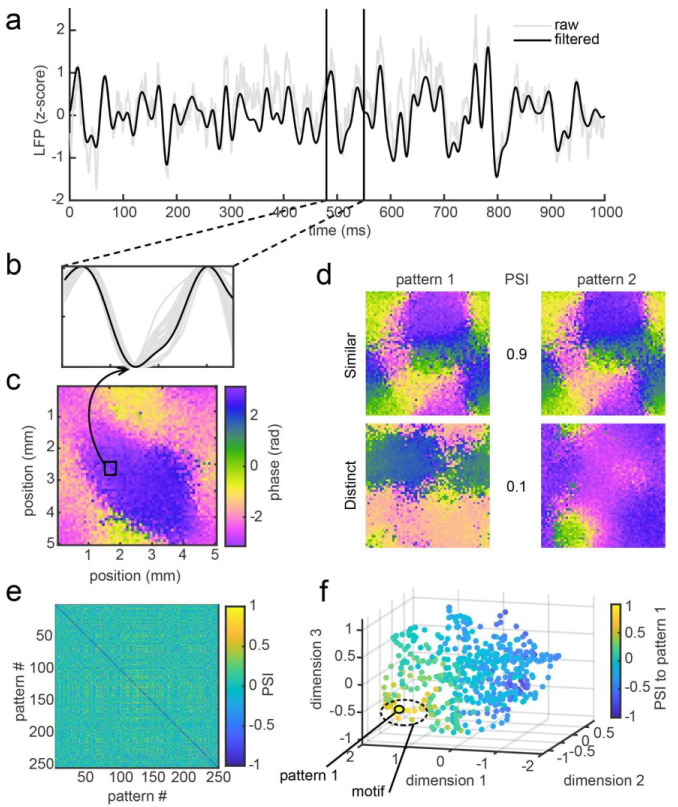
The BPN exhibits repetitions of highly similar iTWs that cluster into motifs. (a) Example trace of model LFP in BPN (raw LFP in gray, 5–50 Hz filtered in black). (b) An individual cycle trough defines a phase pattern across the network (black line: LFP in a; gray lines adjacent model LFPs). (c) Phase pattern across the entire network at the time indicated by the black arrow in b. Box indicates location of LFP in b. (d) 2 pairwise comparisons of PSI with a highly similar pair (top, PSI = 0.9), and a pair with low similarity (bottom, PSI = 0.1). (e) Pairwise PSI values across a subset (N = 250) of phase patterns. Yellow indicates repetitions of highly similar iTWs. (f). Clustering of PSI for iTWs to pattern 1 in d. The hot spot in the lower left corner shows a cluster of highly similar iTW patterns which we group as a motif (dashed ellipse) based on the number of repetitions within a threshold of PSI.

**Figure 3.**
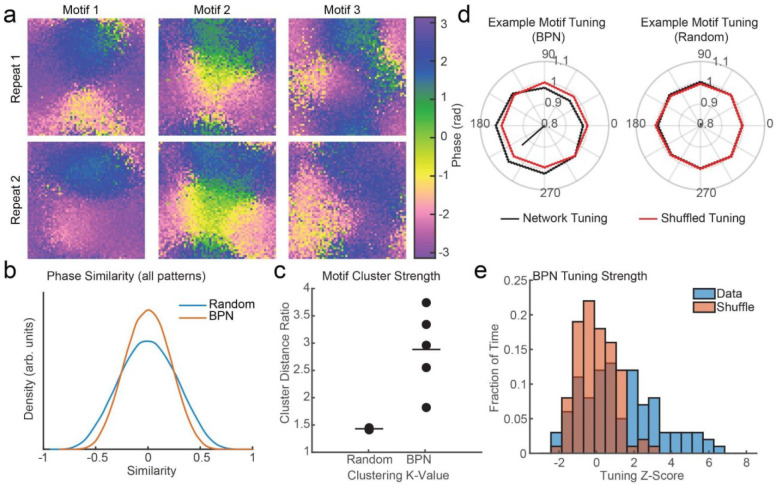
The BPN produces iTW motifs that reflect tuning-dependent spiking activity. (a) Examples of 2 repetitions of highly similar iTWs from 3 identified motifs in a BPN (PSI = 0.71, 0.93, and 0.45 respectively). (b) Distributions of PSI across all phase patterns generated in a random long-range network (blue) or in a BPN (red). The random network shows more overall similarity (positive and negative) than the BPN (N=161868, Kolmogorov-Smirnov test, p = 2.3×10^−153^). (c) The Euclidean distance ratio (distance between clusters v. distance between patterns within a cluster) for identified motifs across networks. Larger values indicate better clustering in motifs. Distance ratios are significantly stronger in the BPN (N=5; distance ratio = 2.88 ± 0.92 C.I.; p = 0.04, one-way ANOVA). (d). Example tuning curves for motifs in the BPN and random network. Black line is normalized spike rates across a motif’s iTWs binned by neuron tuning preference. Red line is the null expectation after shuffling neuron tuning preference. The black arrow is the vector sum of the average binned firing rate. The angle defines the tuning preference of the motif and the length defines the strength of tuning. (e) Distributions of the tuning strength of each phase pattern in the BPN z-scored against the distribution of tuning strengths after shuffling. The BPN (blue) has significantly greater tuning (mean Z = 1.27 ± 0.21 S. E. M.) than expected by chance (red; p = 1×10^−13^ Wilcoxon ranked sum test).

To test this, we first measured iTWs in the BPN. This was done by applying a model^42^ of the LFP which we used to compare iTWs in the original network model with iTWs observed from LFP recordings *in vivo*^35^. We then characterized the similarity in spatial distributions of phase between each pair of iTWs and asked if iTWs formed motifs that were accompanied by feature-selective changes in spiking activity. To measure the similarity between each pair of iTWs, we computed a *Phase Similarity Index* (PSI). PSI values of 1 correspond to iTWs that are identical to one another. Values of 0 correspond to iTWs that are uncorrelated with one another. Values of −1 correspond to iTWs that are identical in spatiotemporal structure but that travel in exactly opposite directions. Figure 2d shows examples of the phase maps of iTW pairs that vary from highly similar (PSI of 0.9) to uncorrelated (PSI of 0.1). We computed PSI values for every pair of iTWs observed in the network simulation, resulting in a phase similarity matrix (Figure 2e). This enabled us to identify iTW motifs—groupings of iTWs that were highly similar to one another based on statistical measures as detailed in Methods (examples in Figure 3a).

We observed that the structured organization of patchy connections in the BPN produced more variable phase patterns than were observed in the random network, as indicated by a narrower distribution of PSI values across all pattern pairs (Figure 3b, blue). The random network model showed more stereotyped activity patterns quantified by broader distributions of PSI values (Figure 3b, red; N=161868 pairs, Kolmogorov-Smirnov test, p = 2.3×10^−153^). We compared the number of iTW motifs from the BPN and random long-range model. The random network produced only two motifs, with each motif encompassing many iTWs (mean = 26). To quantify how well defined the motifs were, we performed spectral clustering on the PSI matrix and looked at the distribution of iTWs across the eigenvectors corresponding to the 3 largest eigenvalues. The motifs in the random network were not well clustered, with the Euclidean distance in the eigenspace between motif centers nearly as large as the average distance between patterns within a motif (distance ratio = 1.43). In contrast, the iTWs of the BPN fell into more motifs (N = 5 with mean = 6.6 patterns; Figure 3c) and the motifs were more clearly differentiated from one another, with a larger ratio of distances among vs. within motifs (distance ratio = 2.88 ± 0.92 C.I.; p=0.04, one-way ANOVA).

We next tested the hypothesis that motifs in the model reflect undriven spiking activity in a feature-selective manner. We binned the neurons in the network according to their assigned tuning preference (9 bins within pi/4 rad of each neuron’s preferred motion direction) and measured the average firing rate of the neurons in each bin during iTWs that were members of a particular motif (spikes included within ± 5 ms of the time of the phase pattern) normalized by the average firing rate of those same neurons across the entire simulation. This produced a tuning curve of modulations in ongoing spike rate as a function of motion direction preference during the motif iTWs (Figure 3d, black). As a control, we performed the same analysis, but after shuffling the tuning preferences of the neurons (Figure 3d, red). The motifs in the model showed a significant degree of tuning preference for motion direction, whereas the motifs observed in the random model showed no tuning bias (N = 5 samples of 5000 units per iTW; Wave Motif Model = 7.04 ± 0.76% S. E. M. modulation v. random = 1.64 ± 0.71% modulation; p = 0.007; Figure 3d). In order to quantify the degree of tuning in the network activity, we calculated the magnitude of tuning (length of the resultant vector for the circular spike-rate modulation) for each phase pattern. These values were z-scored against a null distribution generated by randomly sampling the neurons in each tuning bin 100 times to generate the expected tuning observed by chance. The BPN showed significant tuning compared to the shuffle (mean Z score = 1.27 ± 0.21 S. E. M.; p = 1×10^−13^ Wilcoxon ranked sum test; Figure 3e). These results show that patchy long-range connections can coordinate fluctuations in synaptic activations among like-tuned populations, and this coordination can be read out from repeated patterns of LFP activity in the form of motifs.

To summarize, the BPN makes the testable prediction that feature-selective long-range connectivity gives rise to iTW motifs that reflect the coordination of synaptic activity among like-tuned neurons through their preferred connectivity. While the non-patchy model we had previously developed explained how the spatial alignment of iTWs improve stimulus-evoked gain and perceptual sensitivity in monkeys performing a visual detection task, the BPN further predicts that the depolarization of a particular feature-selective population during an iTW will increase the gain of evoked responses and perceptual sensitivity depending on whether the target also shares the feature preferred by that population.

### Testing BPN Predictions: Motifs in Cortex

**Figure 4.**
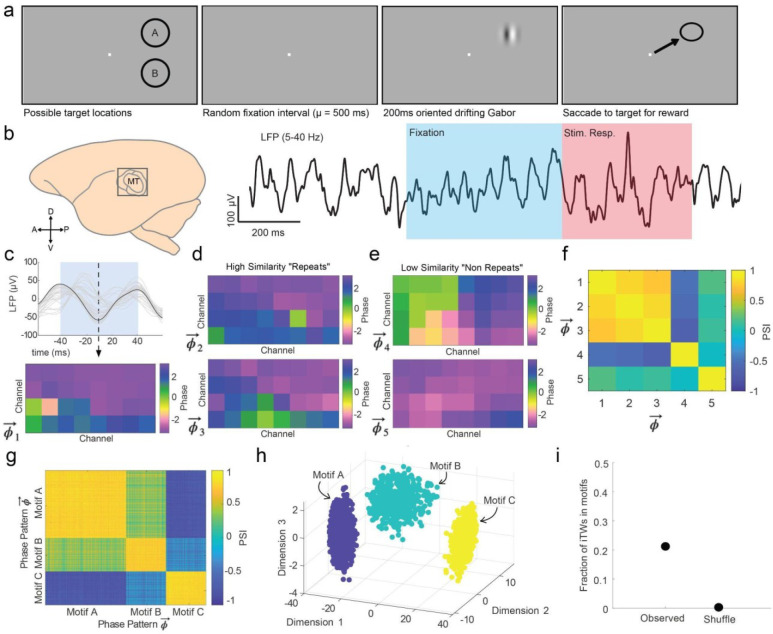
LFP motifs emerge from the ongoing intrinsic activity of Area MT in awake behaving marmosets. (a) Marmosets were trained to report the appearance of an oriented drifting Gabor target at one of two visual locations with a saccade. (b) Multiunit spiking and LFP activity were recorded from the majority of Area MT using chronically implanted Utah arrays. Activity during fixation (blue) and during visual stimulation (red) was segregated for analysis. (c) The upper panel shows an LFP trace recorded on one channel during a cycle (black) and the LFP traces for the remaining channels in gray. Dashed line indicates the time of a candidate iTW phase pattern. The lower panel shows the full phase pattern across each channel at the time of the dashed line. (d) Examples of 2 repetitions of phase patterns with high PSI values to the example shown in (c). (e) Examples of 2 other phase patterns that did not qualify as repetitions due to low PSI values with the pattern in (c). (f) Matrix of PSI among the five patterns in c-e. (g) Matrix of PSI values for all observations of 3 example motifs (A, B, and C). (h) Clustering of the 3 motif patterns in g using the first three eigenvectors of the PSI matrix. (i) The fraction of iTWs that were classified as motifs was significant as shuffling electrode positions and repeating the motif classification analysis failed to yield any motifs.

To test these predictions *in vivo,* we first asked whether iTW motifs could be detected in the spontaneous activity of extrastriate visual cortex. We examined multielectrode Utah array data that we had previously collected from Area MT of two marmosets as they performed a challenging visual detection task^18^ (Figure 4a). In the task, the trial begins when the monkey is shown a marmoset face which draws their gaze to the center of the screen. Upon looking at the face, it is replaced with a fixation point that the monkey has been trained to fixate. After a minimum interval of 300 ms, an additional random delay is drawn from an exponential distribution to yield a flat hazard function for the appearances of the targets. After the delay, a faint Gabor target drifting in one of 6 directions appears at one of two equally eccentric target locations in the upper or lower contralateral visual field aligned with the receptive fields of units recorded on the array electrodes. The monkey had 500 msec from target onset to saccade to the target location for a juice reward. The contrast of the target was titrated such that the monkey detected the target approximately 50 percent of the time. On 10 percent of the trials no target was shown and the monkey was rewarded for holding fixation to the end of the response window. These catch trials helped prevent the monkeys from adopting a guessing strategy and reinforced fixation behaviors.

We divided our data into two periods, the spontaneous ongoing activity during the monkey’s fixation of the blank screen while awaiting the appearance of the target, and the period during the evoked response to the target (Figure 4b). We first looked for motifs in spontaneous LFP patterns during the period of fixation 300 msec after the fixation point appeared (to allow transient activity from visual input and the saccade to fixation to resolve) up until the onset of the target. We filtered the LFP data on each electrode from 5–40 Hz and calculated the Generalized Phase (GP) of the wideband LFP signal. These methods have previously been used to show that the state of iTWs are predictive of network sensitivity and detection performance on the detection task^18^. LFP power < 5 Hz was filtered out to exclude low frequency fluctuations that are associated with slow changes such as changes in arousal^68–70^. LFP power > 40 Hz was excluded to avoid the possibility of bleedthrough of spiking into the LFP, which could potentially lead to spurious spike-LFP coupling unrelated to iTWs^71^. GP is a technique we developed to correct for the issues that can arise from measuring instantaneous phase in a signal that has broad frequency content and is not amiable to the Hilbert Transform, and results in superior quantification of the spike-LFP phase relationship in ongoing spontaneous cortical recordings^72^.

We tested for the presence of motifs similar to the methodology deployed in the model. We first collected all iTWs that occurred during the period of fixation across all trials in our recording sessions (N = 25 sessions in Monkey W, N = 18 sessions in Monkey T). This resulted in 17,468 iTWs in Monkey W and 12,442 iTWs in Monkey T. We observed that, consistent with BPN predictions, some iTWs did exhibit high similarity across the population (Figure 4 c–f). We grouped these iTWs into motifs (4419 or 25% in Monkey W and 2164 or 17% in Monkey T) depending on their degree of similarity and the frequency they occurred. We identified 29 motifs in Monkey W and 42 motifs in Monkey T wherein the phase patterns showed sufficient similarity and repeated more times than expected by chance in our data. Motifs identified in this way form clusters in the low dimensional projection (Figure 4g–h), confirming that these motifs define distinct sets of repeating iTWs.

### Testing BPN Predictions: Feature-Selective Motifs

**Figure 5.**
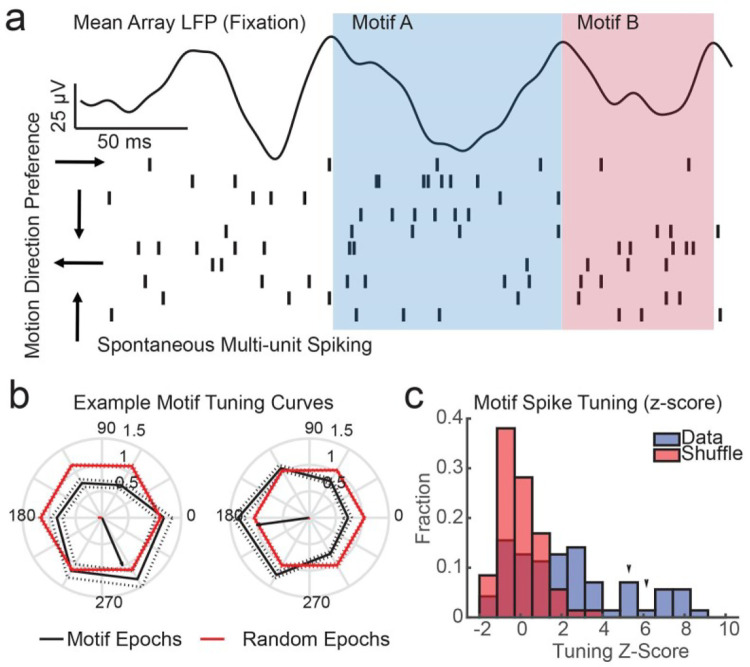
iTW patterns within a motif coincide with modulations in ongoing spiking activity among like-tuned neurons. (a) Demonstration of the calculation of tuning preference for identified LFP motifs. Spikes rates for multi unit activity on each electrode were computed over a window spanning a LFP cycle for each iTW (blue and red shaded regions for motifs A and B respectively) and normalized by the unit’s mean rate over all spontaneous epochs during fixation. These rates were then grouped based on their tuning for motion direction (bins of ± π/3 rad). (b) Example motif tuning curves showing the normalized rates for each motion direction preference during repetitions of a particular motif (black) and the observed curves when arbitrary motifs were randomly generated (red). The tuning direction was defined as the angle of the vector sum of normalized firing rates (black arrow), and significance defined by vector lengths that exceeded the 95th percentile of a randomized null distribution. (c) Histogram showing the distribution of z-scored tuning vector lengths for all identified motifs (blue) and vector lengths for randomly generated motifs (red). Motifs show significantly more tuning than expected by chance (N = 71 motifs across 2 monkeys; mean z = 2.33 ± 0.33 S. E. M.; p = 3.9×10^−9^; Wilcoxon signed-rank test; arrowheads indicated position of examples in b).

The BPN predicts that when iTWs that belong to a particular motif occur there will be a modulation in spiking activity among neurons with common motion-direction preferences, as illustrated in Figure 3d. To test this, we defined intervals for each iTW and computed the spike rate for the single- and multi-unit activity of each channel over that interval, normalized by the average firing rate during fixation (Figure 5a). We then grouped channels based on their preferred direction of motion (6 bins) and calculated how the spontaneous spiking activity varied during a motif’s iTWs as a function of population tuning (Figure 5b). Consistent with the BPN prediction, we observed tuning-dependent spiking across iTWs in a motif.

To define significance for observed tuning, we generated a null distribution of tuned activity modulations by randomly sampling our data 100 times matched to the number of iTWs in each motif. Using these distributions of arbitrarily generated motifs, we recalculated tuning after shuffling channel tuning preferences across our population. We then measured the distribution of chance tuning, defined by the length of the vector sum of normalized spontaneous activity across each motion direction preference, and z-scored each actual motif’s tuning relative to the null distribution. Any motif tuning z-score that exceeded the 95th percentile of the random distribution was classified as significantly tuned. We found that 23/29 motifs in Monkey W and 16/42 motifs in Monkey T were significantly tuned for motion direction preference in spontaneous spiking, and the total distribution across both monkeys was significantly more tuned than expected by chance (mean z = 2.33 ± 0.33 S. E. M.; p = 3.9×10^−9^, Wilcoxon rank sum test; Figure 5c).

Could another mechanism explain the tuning observed during iTWs within motifs? Some degree of tuning might be expected given the known pair-wise spike correlations among like-tuned neurons^73,74^. To control for this we maintained the channel tuning preferences, thus preserving any tuning-dependent spike correlations, but generated random motifs by shuffling iTWs. We found that tuning-dependent correlations alone do yield more tuned spiking modulations than expected from our null distribution (mean z-score = 0.70 ± 0.21 S. E. M.; p = 0.0013, Wilcoxon signed-rank test), but they do not reach the level of tuning observed when iTWs are grouped into defined motifs (p = 0.00022, Wilcoxon signed-rank test). Thus the latent correlations among like-tuned neurons are not sufficient to account for the degree of tuning observed during iTW motifs in our recordings.

### Motifs Modulate Feature-Selective Gain and Perceptual Sensitivity

We next asked whether iTWs in tuned motifs modulate evoked response gain or detection performance for targets on the detection task. Our previous work showed that when iTWs aligned favorable network states with the retinotopic location of visual targets, the responses were stronger and the monkeys were more likely to detect the targets^18^. If these motifs reflect coordination among like-tuned populations, then a similar benefit may occur when the iTW is a member of a motif that is tuned for the features of the target. To test this we first identified trials where there was an iTW within a tuned motif during the onset of the target-evoked response. We did this by measuring the similarity of the first iTW during the evoked-response period (defined as 70–220 ms after target onset) to each member of each motif. We then generated a distribution of evoked-spontaneous iTW similarities, and took as our criterion the 95th percentile of that distribution to define the subset of trials where iTWs at the time of the evoked response belonged to a particular motif.

**Figure 6.**
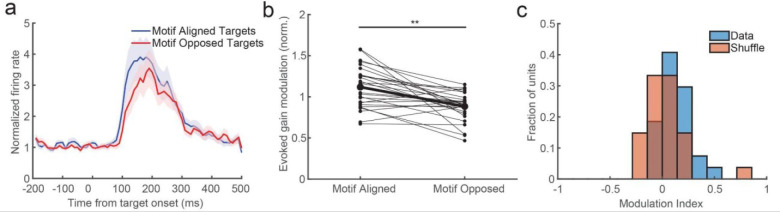
iTWs in motifs modulate neuronal gain in favor of targets aligned with the preferences of that motif. (a) Average evoked spiking responses (normalized to each channel’s baseline firing rate) when the target appeared in the receptive field and was of the channel’s preferred direction. Trials were included in the Motif Aligned average (blue) if the target was within pi/3 rad of the motif’s preferred direction of motion. Trials were included in the Motif Opposed average if the target was within pi/3 rad of the motion direction opposite the preference of the motif (red). The average evoked response was significantly stronger on aligned v. opposed trials (N = 27 channels across both monkeys, 3.22 ± 0.16 S. E. M. v. 2.55 ± 0.15 S. E. M.; p = 0.00044 Wilcoxon signed-rank test). (b) Pairwise gain modulations (normalized to the average evoked response across all trials) for multiunit activity on each channel recorded across both monkeys for the trials described in a. Line thickness indicates the relative number of trials for that unit. Large dots and thick line indicate the population average (1.12 ± 0.05 S. E. M. v. 0.88 ± 0.03 S. E. M.). Units showed significant gain increases on aligned v. opposed trials (p = 0.0002 Wilcoxon signed-rank test). (c) Distribution of spike modulation indices (difference between aligned and opposed divided by their sum) for the data (blue) and a control from randomly generated motifs. iTW Motifs show significantly stronger activity modulations depending on feature alignment than expected by chance (p = 0.017, Wilcoxon rank sum test).

We then asked whether there was any modulation of evoked-gain for motion directions preferred by that iTW’s motif. We first identified all the trials on which the target appeared in each unit’s receptive field and was the motion direction preferred by that unit. We then identified the subset of trials where an iTW motif either preferred the motion direction of the target (within pi/3 rad) or preferred the opposite. We found the evoked spike responses (normalized to each unit’s baseline firing rate) were significantly stronger when targets were aligned to the preference of the motif rather than opposite the preference (N = 27 units; 3.22 ± 0.16 S. E. M. v. 2.55 ± 0.15 S. E. M.; p = 0.00044 Wilcoxon signed-rank test; Figure 6a). To look at how motifs impacted gain across our population of units, we next calculated the evoked-gain modulation for each unit depending on whether the targets were aligned or opposed to the preference of the motifs. We normalized the magnitude of the evoked response under each condition for each unit to the average evoked response across all trials for that unit. Units had significantly stronger responses when targets were aligned to the motif preference compared to opposite targets (1.12 ± 0.05 S. E. M. v. 0.88 ± 0.03 S. E. M.; p = 0.0002, Wilcoxon signed-rank test; Figure 6b). To estimate how much gain modulation we might expect by virtue of random sampling of the data, we calculated a gain modulation index (difference over sum) that quantified how much gain varied between motif-aligned and -opposed trials (Figure 6c). We compared the distribution of modulation indices to those observed when we randomized the trials. We found that the modulation of evoked gain by motifs is significantly stronger than expected by chance (index = 0.11 ± 0.03 S. E. M. v. 0.03 ± 0.04 S. E. M.; p = 0.017, Wilcoxon rank sum test). These results are consistent with the model prediction that motifs reflect periods of excitability among like-tuned populations.

One might wonder if the targets themselves influenced which motifs occurred during the evoked-response period. To answer this question we examined the likelihood of a motif with a particular preference occurring given the feature of the target. Targets at this low contrast did not appear to evoke iTW motifs, as there was no difference in the average PSI between spontaneous patterns and patterns occurring during the evoked response in either monkey (N = over 1,000,000 PSI pairs, H = 0 at α = 0.05; Kolmogorov–Smirnov test), suggesting that the patterns during the evoked response were at most a mixture of both the endogenous network activity and the localized feed-forward activation of the population retinotopically aligned to the target. Further, we tested whether the motion direction of the target was correlated with the tuning preferences of the motifs we observed during the evoked period, as might occur if the target was inducing the motif, and found no relationship between the circular-distance between the target motion direction and the motif direction preference (N = 3384 trials across both monkeys, F = 0.30; p = 0.5848, two-sample Watson-Williams test for equal means). These results suggest iTWs we measure during the evoked-response period are not driven by the appearance of the target but rather are a reflection of the intrinsic network state that the inputs are arriving into.

**Figure 7.**
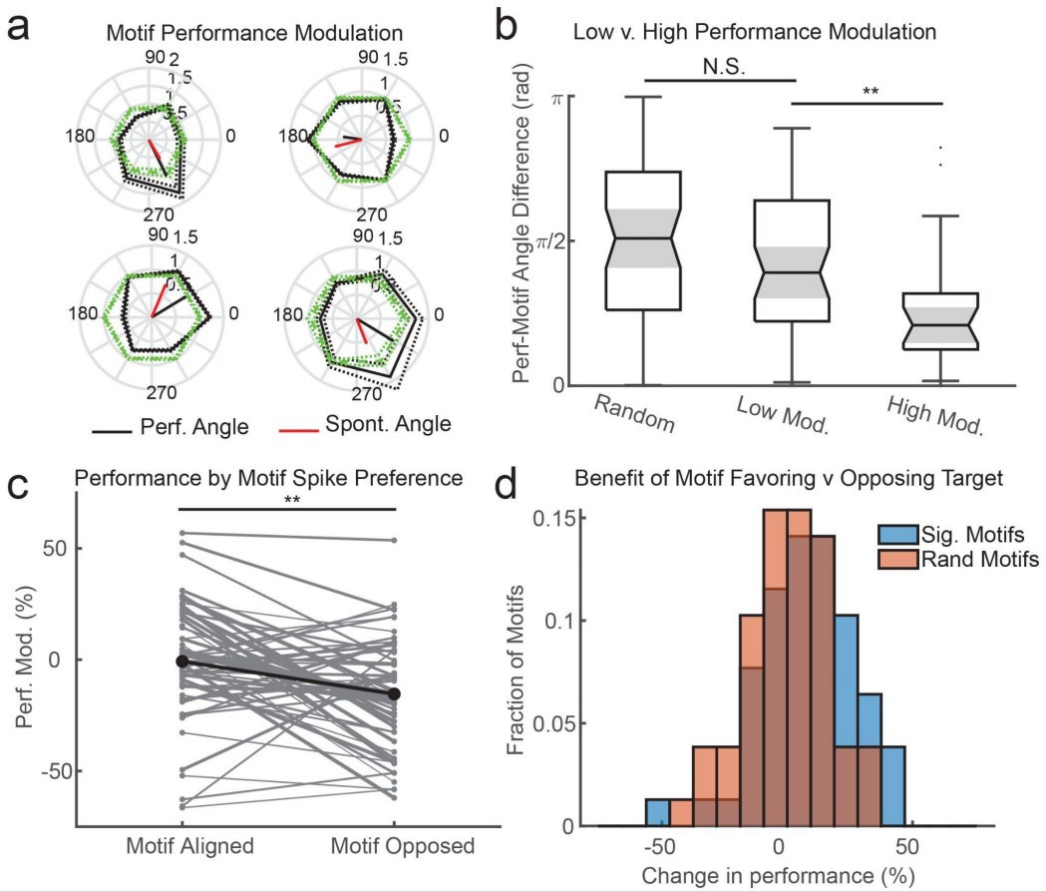
Task performance increases when a target’s motion direction is aligned to the preference of a co-occurring motif. (a) Examples of task performance on trials with motifs during the evoked response. Plotted are the normalized detection performance for each target binned by motion direction (black line) and the 95th percentile confidence interval of 100 iterations of randomly shuffled trials (green line). The angle of the mean circular resultant of the performance distribution (black arrow) tended to point in the same direction as the spontaneous tuning preference of each motif (red arrow). (b) Box-and-whisker plots of the difference between the direction of performance modulation for each motif and the tuning preference of the motif based on spontaneous spike modulations shown in Figure 5. Box boundaries denote upper and lower quartiles, whiskers indicate upper and lower extremes. Dots indicate outliers. Shaded regions indicate bounds of 95% CI of median value. The difference in angle between performance modulation and motif preference was significantly smaller for motifs that showed the high performance modulation (z-score > 1) compared to random motifs or weakly modulated motifs (z-score < 1; p = 0.006 and p = 0.005 respectively, Wilcoxon rank sum test). (c) Paired plot showing the change in monkey performance when the preference of significantly tuned motifs was aligned with (within pi/3 rad) or opposite the motion direction of the target. Black dots and line denote mean across all significantly tuned motifs for each visual field. Asterisk indicates significance at p < 0.001 (Wilcoxon signed-rank test). (d) Histogram showing the distribution of performance modulations of motifs (difference between aligned and opposed; blue) compared to randomly generated but numerically matched motifs (red). Motifs show significantly stronger modulation than the control (p = 0.017, Wilcoxon rank sum test).

Next, we asked whether the observed modulation of evoked-gain by motif preference had any impact on the task performance of the monkey. To test this we calculated the monkey’s detection performance for each direction of motion within an iTW motif (grouped by where the target occurred in the visual field), and normalized it by the monkey’s detection performance for each direction of motion across all trials. We then plotted the change in detection performance for each direction of motion for that motif and z-scored it to a permutation of randomly selected matched trials (Figure 7a). We found many instances where marmosets displayed shifts in task performance specifically for the features preferred by the motifs. Across all the motifs that showed significant spontaneous tuning (N = 39), there were larger shifts in detection performance (measured as the length of the resultant vector of performance changes for each motion direction) than expected from randomly generated matched motifs (z = 0.35 ± 0.16 S. E. M. v. −0.10 ± 0.11 S. E. M.; p = 0.011, Wilcoxon signed-rank test). These results reveal that when iTW motifs occurred during the evoked response there was a significant change in the monkey’s detection probability depending on the feature of that target.

To test whether the performance modulations may just be due to chance, we calculated the difference between the motion direction angle favored by the performance modulation (vector sum) and the motion direction angle favored in the spontaneous spiking as in Figure 5. Across the population of motifs, the angle difference between these two measures was significantly closer to 0 than than expected from randomly shuffling angles. The angle alignment was strongest for motifs that showed the largest performance changes (defined as greater than 1 z-score; mean angular difference = 0.84 rad ± 0.14 S. E. M.), with significantly weaker alignment for random motifs (1.43 rad ± 0.20 S. E. M.) or motifs that produced weak modulations (less than 1 z-score; 1.35 rad ± 0.11 S. E. M.; p = 0.0035 and p = 0.0059 respectively, Wilcoxon rank sum test; Figure 7b). These results show that the performance modulations associated with iTW motifs were specific to the motion directions predicted to be favored by the motif tuning.

Finally, we asked whether the alignment between the tuning preference of a motif and the motion direction of the target showed modulations in detection performance consistent with the gain modulations we saw in the evoked responses. We collapsed trials depending on whether the target motion was aligned or opposed to the motif preference as in the analysis of evoked spike gain. Across the 39 motifs that had shown a significant spontaneous tuning preference we found monkeys consistently performed better when the target was aligned to that preference as compared to opposed (p = 0.0008 Wilcoxon signed-rank test; Figure 7c). This performance modulation was significantly stronger than the distribution of modulations expected from randomly subsampling the data with the same statistics as the motifs (p = 0.017, Wilcoxon rank sum test; Figure 7d). These results are consistent with the predictions of the BPN in which motifs reflect shifts in excitability among sub-networks of neurons linked through preferential tuning-dependent connectivity. Within the model, when these sub-networks are activated, they produce a distinct motif pattern that is predictive of greater depolarization among the like-tuned neurons in that subnetwork. In the data recorded *in vivo*, we find motifs consistent with those in the model that correlate with increases in evoked-gain and better detection performance on the task.

## Discussion

The present findings advance our understanding of the intricate intrinsic dynamics of cortical networks and their role in modulating stimulus-evoked responses and perceptual sensitivity. Using a combination of computational modeling and new analyses of data previously recorded in Area MT of the common marmoset^18^, this study reveals that iTWs exhibit a novel form of feature-selective modulation of neural activity that manifests in both the ongoing undriven and stimulus-evoked responses of neurons. This feature-selective gain modulation influences perception, impacting the perceptual sensitivity of marmosets trained to perform a challenging visual detection task. By demonstrating that feature-selective iTWs emerge naturally from long range feature-selective patchy connections, this study also reveals a new computational role for this prominent anatomical feature of the neocortex. By providing evidence that iTWs are shaped by the architecture of these horizontal fibers, these findings also lend support to the view that iTWs, as measured in the LFP, reflect patterns of spiking activity as they traverse the horizontal fibers that interconnect neocortical neurons.

### Predictions from the Balanced Patchy Network

Our previous model provided evidence that traveling waves, as measured in cortical LFPs, could be explained by the time required for action potentials to traverse the unmyelinated horizontal fibers of the neocortex^35^. The model was also able to explain the modulation of neural response gain and perceptual sensitivity which, in the model, stemmed from shifts in E/I balance that accompany iTWs. However, this model lacked a prominent anatomical feature of the neocortex—horizontal fibers form feature-selective and patchy long-range connections. This anatomical feature provides a test of the proposition that horizontal connectivity shapes iTWs in the cortex and determines their impact on perception. When we grouped iTWs into motifs in the BPN, we found that those iTWs reflect shifts in E/I balance shared across neurons within that motif’s feature-selective subnetwork. iTWs within a given motif thus correspond to feature-selective modulation of ongoing spiking activity.

### Testing BPN Predictions

We analyzed data previously recorded from Area MT of two common marmosets as they engaged in a task that required them to detect a faint motion stimulus presented at perceptual threshold. We found support for the predictions of the BPN. First, we found evidence that iTW motifs can be statistically differentiated and more than half of these motifs (39/71, 55%) were accompanied by feature-selective regulation of ongoing spiking activity. Second, and consistent with earlier model predictions, we found that this feature-selective form of gain control also applied to the spiking response that was evoked by the target. Finally, and consistent with the BPN, we found that monkeys performed better in detecting the target if the direction of stimulus motion was aligned with the feature preference of a coincident motif. That is, motifs that upregulate the gain of neurons that preferred a particular direction of motion were associated with improved perceptual sensitivity to targets moving in that direction.

Since these motifs were defined during the ongoing spiking activity when the monkey fixated a fixation point at the center of an otherwise empty screen, the motifs could not be attributed to waves driven by the appearance of a visual stimulus or eye movements. Control analyses also showed that the pattern of feature-selective regulation of gain could not be explained as stemming from correlated variability among similarly tuned neurons, nor are the feature-selective gain modulations due to the appearance of the target generating particular wave motifs. These results thus support the BPN and support the more general proposition that iTWs emerge, in part, from spikes traversing the horizontal fiber networks in the neocortex.

### Role of Patchy Horizontal Projections

Long range patchy connections have been observed in multiple cortical areas, including visual areas V1^44–46^, V2^47–49^, MT^47,50^, IT^51,52^, PFC^53^ and motor cortex^54^. In V1 of mammals with orientation maps including cats^75–77^, monkeys^78–80^ and tree shrews^46^, they play out in periodic patterns across the cortical surface, connecting neurons with similar orientation preference. It has been suggested that they may play roles in multiple functions including modulation of center-surround interactions^81,82^ and the perception of continuity in visual patterns^83,84^. However, given their ubiquity across areas and species, it seems likely that they may play a broader role in cortical computation^85,86^. The present findings suggest that they serve to coordinate synaptic activity impacting neural and perceptual sensitivity in a feature-selective manner.

### Limitations of the current study

Due to constraints in the construction of the model and the experimental design of our electrophysiological recordings and behavioral tasks, there are some limitations to the inferences that follow from these results. First, the BPN does not include any contribution of thalamocortical, cortico-cortical, or neuromodulatory input that likely contribute to the moment-to-moment spatiotemporal content of cortical LFPs *in vivo*. Therefore we do not have any mechanistic predictions from the model regarding the role those input sources might play in the formation of motifs. In addition to the motifs that are predicted to stem from horizontal patterns of connectivity, motifs could also come from these other sources of input. This could help explain why nearly half of the motifs we identified were not tuned for motion direction.

The presence of untuned motifs could also reflect, to some extent, tuning preferences for features other than the one feature that varied in our experiment: motion direction within the plane of the computer monitor. MT neurons are tuned for multiple motion features, such as speed^87^, motion coherence^88,89^, and disparity and motion in depth^90–92^, and preferences can vary spatially within an MT receptive field^93^. As we did not vary other features in the present study, we are unable to resolve whether these untuned motifs may in fact be tuned for these or other features encoded by MT. Another issue in identifying tuned motifs is that our classification is limited due to statistical sampling. For example, the lower number of significantly tuned motifs in Monkey T may be attributable to the fact that fewer recordings were made in this animal, which resulted in broader null distributions and therefore higher criteria for determining statistical significance. Larger data sets recorded in experiments that vary more stimulus features may identify additional feature-selective motifs.

While we found that some motifs in Area MT are tuned for motion direction, other areas (e.g. V1) are also tuned for similar features such as orientation and are directionally selective and could therefore contribute to the monkey’s detection performance. As we did not record from other cortical areas, we are unable to determine the relative contribution of motifs in MT to task performance. Further, we did not manipulate iTWs or motifs in the present study. We are thus unable to draw a causal inference between the state of iTWs in MT and the monkey’s detection performance. Our interpretation of the causal connection between iTW motifs and behavior, and their dependence on patchy connections, stems from the causal role of patchy connections in forming feature-selective iTW motifs in the model and the mechanism of synaptic coordination through anatomical connectivity.

Finally, our results are limited to the conditions under which the recordings were obtained: with fixating monkeys performing a detection task at perceptual threshold. While we have previously observed that iTWs persist while monkeys view natural scene images^18^, we are currently unable to determine whether the feature-selective motifs reported here would be observed under more naturalistic viewing conditions. Nor can we know from these findings how motifs modulate performance requiring sensory discrimination.

### Potential for Future Research

These findings set the stage for new research directions including studies examining neural underpinnings of iTWs and their broader role beyond regulating perceptual sensitivity. First, while we have focused entirely on the roles of intra-areal horizontal fibers in shaping iTWs, it seems likely that inter-areal projections may also modulate iTWs. Traveling waves across multiple brain areas have been observed in brain-wide calcium imaging in rodents^94,95^ as well as fMRI^96,97^, EEG^98–100^, and intracranial ECoG studies in humans^28^. While the properties of these waves (size, speed, spectral content) are in many ways distinct from the iTWs studied here, and may therefore have different mechanisms and serve different functions, it will be of interest in future work to determine whether iTWs are coordinated across brain areas, potentially creating windows of communication across cortical areas.

Second, while this and our prior work^18^ has shown that iTWs modulate perceptual sensitivity, a key open question is whether iTWs can be adjusted to meet the information processing needs of a particular task. It may be, for example, that iTWs regulate gain differently depending on the perceptual task at hand, regulating the gains of one population of neurons in the context of a detection task and another population to support sensory discrimination. If so, what are the mechanisms that flexibly induce these changes? A related question is whether the deployment of attention, a source of gain-dependent changes that improve sensitivity and discrimination, plays a role in regulating iTWs.

Finally, recent theoretical work from our group has found that recurrently connected networks of neurons that produce iTWs can also encode complex spatiotemporal patterns of activity as they unfold in time^101^. We have shown that such networks can encode patterns on the order of complexity of dynamic natural scenes. As these networks learn particular sequences of events in the natural world, the statistical dependencies of dynamic natural scenes may become embedded in the synaptic weights of the network. This enables it to re-ignite entire naturalistic sequences from their own internal dynamics, when triggered by an appropriate sensory input. We speculate that the feature-selective iTW motifs studied here may reflect just a subset of the patterns that are encoded in the synaptic connectivity of the cortex and that, as we come to understand the full complexity of the patterns that connect neocortical neurons to one another, it will become clear that they have as yet unappreciated computational potential.

In conclusion, this work is a step forward in our understanding of the mechanisms and consequences of spatiotemporally structured activity patterns in cortical networks. These results are consistent with the theory that spikes traversing unmyelinated horizontal fiber networks in cortex produce iTWs that can modulate spiking activity and perceptual sensitivity. The anatomical connectivity of the horizontal fiber network, particularly the patchy connections among like-tuned domains, shapes iTWs into distinct patterns that, within our model, are a reflection of the differential activity among these subnetworks. Fluctuations in the activity of these populations can impact the processing of sensory information, resulting in moment-to-moment changes in spiking responses and perceptual sensitivity in monkeys performing a challenging visual detection task. This work supports the view that iTWs measured in LFP patterns are a reflection of synaptic activity shaped by the anatomical organization of recurrent cortical networks.

## Materials and Methods

### Network Model Simulations

We simulated spiking activity in network models using the specialized program NETSIM (available at http://mullerlab.github.io) previously used to test the sufficiency of horizontal fiber connectivity in the generation of intrinsic traveling waves. Each network model used here consists of N = 450,000 leaky integrate-and fire (LIF) neurons. The excitatory neurons $N_{e}=0.8\;N$, consist of indices $1,2,\ldots ,N_{e}$, where $N_{e}$ is a square number, arranged uniformly on a two-dimensional grid. Similarly, the $N_{i}=0.2N$ inhibitory neurons, of indices $N_{e}+1,N_{e}+2,\ldots ,N$, where $N_{i}$ is also a square number, arranged uniformly on a two-dimensional grid. Both grids have side length L = 5 mm and they are concentric, together forming a two-dimensional sheet of the N neurons. Each neuron in the network forms K=1000 connections with other neurons in the network. The membrane potential $V^{\left(i\right)}$ of the i^th^ neuron evolves according to the equations:

$$C_{m}\dot{V}=G_{L}(E_{L}–V^{\left(i\right)})+g_{e}^{\left(i\right)}(E_{e}–V^{\left(i\right)})+g_{i}^{\left(i\right)}(E_{i}–V^{\left(i\right)}),\;\tau_{\left\{e,i\right\}}{\dot{g}}_{\left\{e,i\right\}}^{\left(i\right)}=–g_{\left\{e,i\right\}}^{\left(i\right)},$$

where $C_{m}$ is the membrane capacitance, $G_{L}$ is the leak conductance, $E_{L}$ is the resting membrane potential, $\tau_{\left\{e,i\right\}}$ are the excitatory and inhibitory synaptic time constants, $g_{\left\{e,i\right\}}^{\left(i\right)}$ are the time-dependent synaptic conductances of the i^th^ neuron, and $E_{\left\{e,i\right\}}$ are the reversal potentials for excitatory and inhibitory synaptic transmission, respectively.

When $V^{\left(i\right)}$ exceeds threshold $V_{T}$ at time $t_{S}$, the following spike and reset conditions occur:

$$\begin{array}{ll} & V^{\left(i\right)}\mapsto V_{r},\\ & t_{n}=t_{s}+\tau^{\left(i,j\right)},g_{\left\{e,i\right\}}^{\left(j\right)}\mapsto g_{\left\{e,i\right\}}^{\left(j\right)}+G_{\left\{e,i\right\}}\forall j\in [1,K], \end{array}$$

where $V_{r}$ is the reset potential, $t_{n}$ is the time at which the postsynaptic neuron receives its input following axonal and synaptic delays, $G_{\left\{e,i\right\}}$ are the excitatory and inhibitory synaptic weights, $g_{\left\{e,i\right\}}^{\left(j\right)}$ are the excitatory and inhibitory conductances of postsynaptic neuron j≠i, respectively, and K is the number of postsynaptic targets of neuron i. Immediately after neuron i spikes, it undergoes a refractory period of $t_{r}$ where the membrane potential is not updated.

Axonal conduction delays increased linearly with distance between pre- and postsynaptic cells:

$$\tau^{\left(i,j\right)}=\tau_{s}+\frac{d^{\left(i,j\right)}}{v_{c}^{{\left(i,j\right)}^{'}}}$$

where $\tau^{\left(i,j\right)}$ is the delay from neuron i to neuron $j,\tau_{s}$ is the same delay representing synaptic vesicle release as in the random network, $d^{\left(i,j\right)}$ is the Euclidean distance between neurons i and j, and $v_{c}^{\left(i,j\right)}$ is the axonal conduction speed for the connection from neuron i to neuron j. All distances were calculated taking two-dimensional periodic boundary conditions into account, effectively wrapping the network onto a toroidal topology.

Instead of initializing self-sustained activity through a “kick” of external Poisson input spikes, which may induce trace activity correlations, we recorded the state variables of a self-sustained network, including membrane potential $V^{\left(i\right)}$ and conductance $g_{\left\{e,i\right\}}^{\left(j\right)}$, after a long period (10 seconds) of simulated self-sustained activity. Taking these distributions as a steady state, we then used the Gaussian approximation (mean and variance) to initialize the membrane potentials and conductances with randomly drawn values in the simulations thereafter. After starting the simulation with these initial conditions, networks with approximately balanced excitation and inhibition exhibit self-sustained, irregular spiking activity. Each simulation ran for 1.2 seconds, from which we eliminated the first 200 ms from our analysis in case of residual initialization artifacts.

In the original local-only network, connections between neurons (excitatory and inhibitory) were randomly drawn from an isotropic two-dimensional Gaussian probability distribution of zero mean and standard deviation σ = 600μm in either dimension. In the random long-range network, 10 percent of the connections are rewired randomly without regard for spatial distance. In the BPN, the connection probability for excitatory neurons was the weighted sum (90/10) between the two-dimensional Gaussian used in the local-only network and a probability map generated by calculating the circular distance between each neuron’s assigned tuning preference based on a von Mises distribution $P_{c}=\frac{exp(kcos(\phi ))}{2\pi }$ where k is the concentration parameter of the von Mises distribution, ϕ is the circular distance between each neuron’s tuning preference. A value of k=1.25 was used for the main results presented here and were qualitatively similar for a range of values. The value of $P_{c}$ is normalized to have a maximum of 1. The inhibitory neurons were spatially restricted such that probabilities beyond the extent of the two-dimensional Gaussian were set to zero. For each neuron, 0.8K connections to excitatory neurons were randomly selected based on the spatial weights of the probability map corresponding to excitatory or inhibitory projections using the *randsample* function in MATLAB. 0.2K connections to inhibitory neurons were made at the locations of the projections to excitatory neurons.

The motion direction tuning preference of each neuron is assigned based on a topographic map generated to emulate the pinwheel tuning organization of primate cortex. First, a single square pinwheel of L = 500 μm is generated with each neuron index within the pinwheel assigned a tuning preference based on its distance and angle from the pinwheel center. In order to uniformly sample the circular distribution of angles across a square two-dimensional network, we created a weighted distribution of angles where the probability density of the angles was counterphase to the density of angles present when a square is inscribed in a circle. This was done by randomly sampling a sinusoidal distribution $P_{\theta }=sin(\theta )\times (1–\frac{1}{\sqrt{2}})+1$ where θ is the circular distribution of angles from 0 to 2π shifted by $\frac{\pi }{2}$. After randomly sampling the resulting angle distribution using the *randsample* function in MATLAB, the new distribution is then ordered from 0 to 2π and each neuron at cartesian position (x,y) within the pinwheel assigned the tuning based on the polar position (r,θ). To ensure each angle is equally represented across the neuron population, we binned θ into 100 values between 0 and 2π and counted the frequency of each angle. We deployed a shuffling algorithm where counts over/under the desired uniform value were randomly replaced with an angle of an adjacent bin until all bins had the same count. As a result, for an individual pinwheel consisting of 60×60 excitatory neurons (500μm × 500μm) each of the 100 tuning bins had a count of 36 neurons. This pinwheel was then mirrored across its horizontal and vertical axis to create 4 pinwheels with L = 1 mm, and mirrored again to produce 16 pinwheels with L = 2, and again to L = 4 and again to L = 8. The angle at indices $1,2,\ldots \sqrt{N_{e}}$ for each side corresponded to each excitatory neuron in the network and the angle at indices $2,4,\ldots \sqrt{N_{e}}$ to each inhibitory neuron.

### Model of the LFP

In order to generate an estimate of the network LFP for comparison with *in vivo* LFP recordings, we utilized a previously developed proxy for the LFP^42^. The LFP estimate λ(t) is computed as a weighted sum of the excitatory and inhibitory synaptic currents $I_{e}$ and $I_{i}$ across m excitatory cells in each non-overlapping 10 × 10 neuron pool across the spatial extent of the network:

$$\lambda \left(t\right)=\sum_{j=1}^{m}\;I_{e}^{\left(j\right)}\left(t–\tau \right)–\alpha \sum_{j=1}^{m}\;I_{i}^{\left(j\right)}\left(t\right)$$

Where τ=6ms,α=1.65, and m=100 excitatory cells. These values of τ and α were found by the authors to agree with the LFP generated from a three-dimensional model of spatially extended multi-compartment model neurons analogous to the laminated cortical network. Here we computed the LFP using the pooled excitatory and inhibitory synaptic conductances and the driving force between the mean pooled membrane potential and the synaptic reversal potential to calculate the average current in each pool. The LFP proxy was thus independent across each pool, unlike cortical recordings where LFP signals show varying frequency dependent scales of spatial integration.

### Electrophysiological Recordings

The data analyzed in this work was collected from two marmoset monkeys (*Callithrix jacchus*; one male: monkey W and one female: monkey T). These data were previously analyzed as described in Davis et al. *Nature* 2020. Each monkey was surgically implanted with a headpost for head stabilization and eye tracking. A craniotomy and duratomy was made over Area MT (stereotaxic coordinates 2 mm anterior, 12 mm dorsal). An 8×8 (64 channel, monkey W) and 9×9 with alternating channels removed (40 channel, monkey T) Utah array was implanted using a pneumatic inserter. The electrode spacing was 400 μM with a pitch depth of 1.5 mm. The craniotomy was closed with Duraseal (Integra Life Sciences, monkey W) or Duragen (Integra Life Sciences, monkey T), and covered with a titanium mesh embedded in dental acrylic. All surgical procedures were performed with the monkeys under general anesthesia in an aseptic environment in compliance with NIH guidelines. All experimental methods were approved by the Institutional Animal Care and Use Committee (IACUC) of the Salk Institute for Biological Studies and conformed with NIH guidelines (protocol 14–00014).

Multielectrode recordings from the Utah arrays were made via 2 32-channel Intan RHD 2132 headstages connected to an Intan RHD2000 USB interface board (Intan Technologies LLC, Los Angeles, USA) controlled by a Windows computer. Data were sampled at 30 kHz from all channels. Digital and analog signals were coordinated through National Instrument DAQ cards (NI PCI6621) and BNC breakout boxes (NI BNC2090A). Neural data was broken into two streams for offline processing of spikes (single-unit and multi-unit activity) and LFPs. Spike data was high-pass filtered at 500 Hz and candidate spike waveforms were defined as exceeding 4 standard deviations of a sliding 1 second window of ongoing voltage fluctuations. Artifacts were rejected if appearing synchronously (within 0.5 ms) on over a quarter of all recorded channels. Segments of data (1.5 ms) around the time of candidate spikes were selected for spike sorting using principal component analysis through the open source spike sorting software MClust in Matlab (A. David Redish, University of Minnesota). Sorted units were classified as single- or multi-units, or noise and single units were validated by the presence of a clear refractory period in the autocorrelogram. Units classified as noise were eliminated from analysis and single and multi-unit spikes recorded on the same channel were combined to give a single channel spike count. LFP data was low-pass filtered at 300 Hz and down-sampled to 1000 Hz.

### Detection Task

Marmosets were trained to enter a custom-built marmoset chair that was placed inside a faraday box with an LCD monitor (ASUS VG248QE) at a distance of 40 cm. The monitor was set to a refresh rate of 100 Hz and gamma corrected with a mean gray luminance of 75 candelas/m^2^. The marmosets were headfixed by the headpost for all recordings. Eye position was measured with an IScan CCD infrared camera sampling at 500 Hz. The MonkeyLogic software package developed in MATLAB (https://www.brown.edu/Research/monkeylogic/; https://monkeylogic.nimh.nih.gov/)^102^ was used for stimulus presentation, behavioral control, and recording of eye position.

The marmosets were trained to saccade to a marmoset face to initiate each trial. Upon the gaze arriving at the face, it disappeared and was replaced with a white fixation point (0.15 DVA). The marmosets held fixation on the fixation point (1.5 visual degree tolerance) for a minimum duration (400 ms monkey W, 300 ms monkey T) awaiting the appearance of a drifting Gabor target (4 DVA diameter; appearing 6–7 DVA eccentricity at 1 of 2 equally eccentric locations in the visual field contralateral to the recording array). The Gabor could drift in one of 8 equally spaced radial directions for monkey W or 6 directions for monkey T (Gabor spatial frequency: 0.5 cycles per degree; temporal frequency: 10 cycles per second). The target contrast was titrated to a value that each monkey detected approximately 50 percent of the time (1–2% Michelson contrast). The onset time of the target (after the minimum delay) was drawn from an exponential distribution (mean 200 ms) to yield a flat hazard function. After the delay, the target appeared for 200 ms and the monkey had 500 ms from target onset to saccade to the location of the target for a juice reward. Early fixation breaks (defined by the excursion of the eye position from the fixation window) were excluded from analysis. High contrast probes (10% Michelson contrast) randomly occurred on 10 percent of trials. If the monkey’s performance on these easy target trials was below 70% within a session that session was excluded from analysis. On 10 percent of the trials no target was shown and the monkey was rewarded for maintaining fixation until the end of the response window. These “catch” trials served to reinforce fixation behaviors and suppress guessing strategies. The monkeys performed well on these trials (monkey W: 71% correct reject; monkey T: 73% correct reject) suggesting they did not adopt a guessing strategy.

### Receptive field mapping

MT receptive fields were mapped by calculating the spike-triggered average from the reverse correlation of visual probes presented on the computer monitor. The marmoset began a mapping trial by fixating an image of a marmoset face. After holding fixation for 200 ms, a drifting Gabor target (50 percent Michelson contrast; spatial frequency: 0.5 cycles per degree; temporal frequency: 10 cycles per second; drifting in one of 12 (monkey W) or 6 (monkey T) possible directions) appeared at a random location on the screen. After 200 ms the Gabor disappeared and, after a random delay drawn from an exponential distribution (mean delay 30 ms) reappeared at another random location. The processes repeated until the monkey broke fixation (defined as an excursion from the fixation zone beyond 1.5 d.v.a.). The monkey received a juice reward proportional to the number of probes presented on the receptive field mapping trial. We calculated both the spatial location of the receptive field of single and multi-unit activity on the Utah array as well as the tuning curves for the preference for motion direction of the probes. The average evoked response to each direction of motion was normalized by the mean overall response, and the angle of motion preference was defined as the angle of the vector sum of each direction’s normalized response magnitude (measured from 50–200 ms after target onset). Based on receptive field locations, the top 32 channels of Monkey W’s Utah array were excluded from analysis as these channels were likely to be outside of Area MT.

### Spontaneous Activity Analysis

Spontaneous data were analyzed from the period of fixation preceding the onset of the target evoked response (conservatively estimated to be after 50 ms) and excluding the initial 200 ms following fixation initiation. Candidate LFP fluctuations for defining motifs were selected after filtering the raw LFP from 5–40 Hz (4th order Butterworth forward-reverse filter using the *filtfilt* function in MATLAB). We then calculated the *Generalized Phase* (GP) of this wideband signal using previously described methods for calculating phase of signals with relatively broad spectral content. GP represents an advancement over the standard Hilbert Transform as it corrects for the breakdowns that can occur when calculating the instantaneous phase of a signal with broad spectral content. Individual cycles in this wideband filtered data form candidate spatiotemporal patterns for defining iTW motifs. These protocycles were identified by taking the mean phase over a 3×3 region of electrodes near the retinotopic location of visual targets on the array. The cycle window was defined as the period from 0 radians to 0 radians (within a tolerance of 0.2 rad) and the test time defined as the moment that was closest to ± pi radians. This anchors the comparison between candidate patterns as otherwise two identical trajectories might be classified as different simply because they are out of phase with one another in time.

### Defining Motifs

To quantify the similarity between iTWs we computed a Phase Similarity Index (PSI) which measured the circular-circular correlation between each pair of the $N_{p}$ phase patterns observed in the data. This resulted in an $N_{p}\times N_{p}$ correlation matrix, C, where $c_{j,k}$_,_ denoted the similarity between phase patterns $j,k\in \left[1,N_{p}\right]$.

A similarity threshold S∈[–1,1] was then defined such that phase patterns with PSI above S were considered to be examples of patterns belonging to the same motif. In our main analyses, S ranged from 0.55–0.8, and effects of varying this parameter are explored in the following section (Effects of Parameters).

The correlation matrix C was then binarized by the similarity threshold S. The sum of elements in row j of $C,r_{j}=\sum_{k=1}^{N_{p}}\;c_{j,k}$, therefore corresponds to the number of phase patterns with above threshold similarity to phase pattern j. We refer to these patterns as “repeats” of pattern j. We define “high repetition” phase patterns to be the patterns that repeat at least R times more than average, where R>1 is a chosen “repetition threshold”. Specifically, phase pattern j is a “high repetition” pattern if $\frac{r_{j}}{{\hat{r}}_{j}}>R$, where ${\hat{r}}_{j}$ denotes the mean number of repeats across phase patterns $j\in \left[1,N_{p}\right]$. In our main analyses, R ranged from 3–5, and effects of varying this parameter are explored in the following section (Effects of Parameters). These high repetition phase patterns were then used to define motifs.

Let $\left\{h_{j}\right\}$ denote the set of high repetition phase patterns, with each pattern $h_{j}$ corresponding to a set $m_{h_{j}}$ of $r_{j}$ repeats given by the phase patterns corresponding to nonzero elements in row j of ρ. Starting with $h_{1}$, we define the first motif to be $m_{1}=m_{h_{1}}$. If $h_{2}\notin m_{h_{1}}$, then $h_{2}$ defines a new motif, $m_{2}=m_{h_{2}}$. Otherwise, $h_{2}$ does not define a new motif, and we move on to $h_{3}$. This process defines each new motif to be the set of repeats of a high repetition phase pattern, provided that phase pattern does not already belong to a motif, thus reducing the overlap between motifs.

### Effects of Parameters

Our main analyses used parameter values: S=0.55,R=3 for motifs in the model, S=0.8,R=5 for NHP W, and S=0.65,R=5 for NHP T. The use of different criteria between monkeys (and in the model) was due to differences in sampling based on the number of observations and the number of electrodes in MT in each monkey. Specifically, these values vary due to differences in the distributions of PSI values observed in the data, which resulted from differing numbers and placements of electrodes in Area MT. Fewer high PSI values were observed in the model compared to recorded data due to the increased number of samples (60×60 model LFPs), and a lower similarity threshold was therefore required for the model. Similarly, a lower threshold was required for NHP T due to larger spacing between recording electrodes which resulted in lower PSI values overall. Importantly, the presence of motifs did not depend on the specific use of these parameters. We explored a wide range of values for both S and R to understand the impact of these parameters on the clustering of resulting motifs. When determining which parameters to use for the final analysis, we included in our criteria the need for sufficient motifs and numerous iTWs in each motif to make meaningful measurements of their effects on spiking and task performance.

### Identifying phase patterns as traveling waves

Phase patterns were only included in further analysis if it was identified as a traveling wave based on a statistical approach previously used to detect spontaneous traveling waves in noisy multichannel recordings^18^. The wave detection technique first finds the channel location that maximizes the divergence of the phase gradient of the scalar phase field across all electrodes. This captures the point from which activity is flowing outward on this phase pattern. Next, with this putative source point found, the algorithm quantifies the circular-linear correlation of phase with distance: $\rho_{\phi d}\in [–1,1]$ with distance calculated from the source point. Finally, the statistical significance of this correlation is compared against a null distribution of $\rho_{\phi d}$ generated by randomly shuffling the spatial locations of the phase values across the array. From the observed chance distributions of $\rho_{\phi d}$ in our recordings we take as a threshold for meaningful wave-like structure a value of $\rho_{\phi d}=0.3$. Motif patterns below this threshold were excluded from subsequent analysis.

### Motif Tuning

We identified the tuning preference of spiking activity on each channel (combined single- and multi-unit) based on the previously described receptive field mapping. Tuning preference was defined as the angle of the mean circular resultant of average spiking responses to RF probes of each direction of motion. Channels with a mean circular resultant length less than 0.05 were classified as showing no tuning preference and were excluded from analysis (7/40 Monkey T; 1/32 Monkey W). In order to test for motif tuning, spiking activity was grouped into 6 motion direction bins. For each pattern within a motif, the spiking activity during an iTW cycle (as defined above under *Spontaneous Activity Analysis*) was counted for each channel and normalized to the average spike rate during all spontaneous activity for that channel. This produced a circular vector of motif-dependent tuning modulation, and the angle of the mean circular resultant defined the motion direction preference. An identical analysis was performed on 100 permutations of randomly generated motifs that were matched in the number of patterns belonging to each motif. Tuning significance was defined for motifs where the length of the mean circular resultant of the tuning modulation exceeded the 95th percentile of the null distribution of vector lengths from the randomly generated motifs. Motifs that did not show significant tuning were excluded from subsequent analyses.

### Motif Modulation of Evoked Responses

In order to classify the evoked responses to target in the detection task as being members of identified motifs, we identified the first cycle as described in *Spontaneous Activity Analyses* that occurred during the evoked response (defined as between 70–220 msec after target onset). We excluded catch trials, fixation breaks, and high contrast probe trials from our dataset. We then calculated the average PSI value between each iTW during the evoked response to every pattern that was a member of an individual motif. This was done across all motifs we had identified in the spontaneous activity. We then assigned each trial to a motif if it showed greater average similarity to the members of that motif than the 95th percentile of all trials. As a control, we then randomly assigned trials to motifs matching the counts that we observed based on the PSI values. These motif-similar and motif-random trials served as the pools underlying the subsequent analyses.

Next, we identified each channel that showed a significant evoked response to the appearance of the target at one of the two possible locations. For each of these channels, we identified the subset of trials where the target appeared in the channel’s receptive field and was of the preferred direction of motion (within π/3 rad) based on that channel’s receptive field mapping. We calculated that channel’s average evoked response across this subset of trials. We performed the same calculation on the subset of motif-similar trial where that channel’s preference was aligned with (within π/2 rad), or opposite to (more than π/2 rad), the tuning preference assigned to the motif based on the Motif Tuning analysis. These formed two collections of trials, motif-similar trials that prefer the target, and motif-similar trials that prefer the opposite target, holding constant the neuronal preference for the target across both collections. The same procedure was performed on the motif-random trials. Motif trials were only included if there were more than 5 trials satisfying these criteria. We calculated a motif modulation index on the evoked gain: $MI_{m}=\frac{R_{a}–R_{o}}{R_{a}+R_{o}}$ where $R_{a}$ is the baseline normalized evoked response on the preference-aligned collection and $R_{o}$ is the baseline normalized evoked response on the preference-opposite collection.

### Motif Modulation of Detection Performance

We first calculated each monkey’s detection performance for each target type at each target location excluding high contrast targets. Then, using the same motif-similar and motif-random trials defined in Motif Modulation of Evoked Responses, we calculated the change in detection performance for each target direction of motion at each location for the motif-similar and motif-random trials for each motif, resulting in 2 performance tuning curves for each motif, one for each target location. For each motif tuning curve we took the length and angle of the mean circular resultant as the strength of tuning and the tuning preference of the motif respectively. To test the significance of each value, a null distribution of lengths for each tuning curve was generated by randomly sampling trials matched to the count of motif-similar trials for each direction of motion and target location 100 times and the observed values were z-scored against this null distribution.

To test whether the spontaneous tuning preference of the motifs were predictive of performance modulations on the detection task, we compared trials where targets were aligned with, or opposite to the tuning preference assigned to the motifs as in the Motif Modulation of Evoked Responses. For each motif’s motif-similar or motif-random trials, we collected all the trials where the target motion was aligned to (within π/3) or opposite (greater than π-π/3) the motif preference assigned as described in Motif Tuning. This was done separately for targets appearing in the upper or lower visual fields. We required that at least 10 observations of aligned motif-similar and opposite motif-similar trials were present to be included in the analysis. We separated motifs that had shown a significant tuning preference (defined as a mean circular resultant length greater than the 95th percentile of the null distribution) from those that did not show significant tuning (less than the 95th percentile). These were defined as strongly or weakly modulated motifs respectively.
